# Preoperative polycythemia may be associated with inferior postoperative outcomes: a retrospective study

**DOI:** 10.3325/cmj.2025.66.186

**Published:** 2025-06

**Authors:** Ivan Krečak, Marija Valovičić Krečak, Iva Lazinica, Marko Lucijanić

**Affiliations:** 1 Department of Internal Medicine, General Hospial of Šibenik Knin County, Šibenik, Croatia; 2 Faculty of Medicine, University of Rijeka, Rijeka, Croatia; 3 University of Applied Sciences, Šibenik, Croatia; 4 Department for Quality Assurance and Improvement of Health Care, General Hospital of Šibenik-Knin County, Šibenik, Croatia; 5 Department of Hematology, Dubrava University Hospital, Zagreb, Croatia; 6 School of Medicine, University of Zagreb, Zagreb, Croatia

## Abstract

**Aim:**

To investigate the influence of preoperative polycythemia on postoperative outcomes.

**Methods:**

We retrospectively reviewed the postoperative outcomes of 1196 elective non-cardiac surgery procedures (minor, 36%; intermediate/major, 64%) performed under general anesthesia at the General Hospital of Šibenik-Knin County, Croatia, between January 1, 2023 and January 1, 2024. Patients were stratified preoperatively as having anemia, normal hemoglobin, or polycythemia. The primary outcome was a 30-day postoperative composite outcome consisting of death, thrombosis, major bleeding, and the need for red blood cell transfusion.

**Results:**

Anemia, normal hemoglobin levels, and polycythemia were recorded preoperatively in 152 (12.7%), 1000 (83.6%), and 44 (3.7%) of patients, respectively. Patients with polycythemia were the youngest, more frequently men and smokers, and had the lowest frequency of prior venous thromboembolism (VTE). Patients with anemia were the oldest and most frequently had comorbidities, cancer, and prior VTE, used anticoagulants, and underwent intermediate/major surgeries. The composite outcome was recorded in 91 procedures (7.6%) and was most frequent in patients with polycythemia (18.2% vs 9.2% vs 6.9%; *P* = 0.016). Patients with polycythemia also most frequently had postoperative bleeding (18.2% vs 7.9% vs 6.5%; *P* = 0.011) and did not need postoperative red blood cell transfusions (*P* = 0.003). The associations of preoperative polycythemia with the postoperative composite outcome and bleeding remained significant in multivariate models adjusted for surgery risk, sex, comorbidities, physical status, and antiplatelet or anticoagulant use. Patients with polycythemia did not experience deaths or thrombotic events.

**Conclusion:**

Patients with polycythemia require comprehensive preoperative assessment. Future studies are needed to investigate the pathophysiological mechanisms underlying the observed effects.

Preoperative assessments are used to identify and optimize patient comorbidities that may lead to complications during general anesthesia and surgery, and after surgery. Perioperative risk is multifactorial and includes patient-related comorbidities, surgery risk, and the type of anesthetic administered. To optimize postoperative outcomes, timely and comprehensive preoperative assessment is needed.

Complete blood cell count is a widely used preoperative test (1), although it is sometimes criticized for not being cost-effective (2). While preoperative anemia is associated with increased postoperative morbidity, mortality, and transfusion requirements (3), it is still unclear whether and to what extent polycythemia may affect postoperative outcomes. Additionally, polycythemia vera (PV), a myeloproliferative neoplasm (MPN) characterized by clonal myeloproliferation and high cardiovascular risk, is still underrecognized and undertreated. Notably, one-fourth of PV patients will experience a thrombotic event during lifetime (4,5). Therefore, these patients should be recognized early to initiate proper treatment. Phlebotomies and low-dose aspirin are sufficient in low-risk PV patients (those younger than 60 years of age and without prior thrombotic event), whereas all other patients additionally receive cytoreduction, usually with hydroxyurea or interferons (3). MPN patients have high postsurgery complication rates (7%-11%), with vascular events and major hemorrhages being the most frequent complications. These events were frequent despite adequate blood cell count control with cytoreduction and the use of antiplatelets and anticoagulants (6,7). PV patients also had more frequent thrombotic and hemorrhagic complication rates after total joint arthroplasty than controls (8).

When it comes to secondary polycythemia (SP), its effects on postoperative outcomes are still unclear. One single-center retrospective study from 1991 showed similar postoperative outcomes in 100 patients with chronic obstructive disease having SP and matched controls (9). Conversely, a more recent study (10) has shown higher postoperative complication rates in SP than PV patients, namely thrombosis (13% vs 3%). Additionally, SP *per se* may not be as benign as previously thought, with the baseline incidence of thrombotic rates being comparable to PV in some studies (11) and its future thrombotic risk resembling low-risk PV (12). Moreover, a large population-based study has demonstrated that erythrocytosis in the general population could be associated with significant cardiovascular morbidity (13).

In recent years, the World Health Organization (WHO) has significantly lowered the diagnostic criteria for PV (hemoglobin >165 g/L for men and >160 g/L for women), and the number of PV investigations increased substantially (14). Notably, one study reported that 3%-4% of the Canadian population would require an investigation of PV based solely on their laboratory values (15). Prior studies investigating the effect of preoperative polycythemia on postoperative outcomes were performed on a small number of patients (24-100 patients) and had a very small number of events, which precluded firm conclusions. Therefore, considering the increasing number of referrals for polycythemia investigations worldwide, we found it relevant and timely to investigate whether preoperative polycythemia may affect postoperative outcomes.

## Patients and methods

### Study design and patient inclusion criteria

This single-center retrospective study was conducted at the General Hospital of Šibenik-Knin County, Šibenik, Croatia. We reviewed the medical records of all patients who had undergone elective non-cardiac and non-neurological surgery under general anesthesia between January 1, 2023 and January 1, 2024. Clinical and laboratory data were collected. Patients who had undergone emergency and ophthalmological surgeries and endoscopy procedures under general anesthesia, and patients younger than 18 years of age were excluded.

Comorbidities were assessed individually and cumulatively according to the age-adjusted Charlson Comorbidity Index (aCCI) (16). The preoperative physical status was assessed according to American Society of Anesthesiologists (ASA) classification (1 = healthy patient; 2 = mild or moderate systemic disease caused by a surgical condition or another pathological process, and medically well controlled; 3 = a severe disease that limits activity but is not incapacitating, 4 = a severe incapacitating disease that is a constant threat to life, 5 = moribund patients not expected to survive for 24 hours) (17).

Surgery procedures were classified based on their perioperative cardiovascular risk as minor (<1%; thyroid/parathyroid, breast, meniscectomy, minor urological and minor gynecological surgeries, plastic and reconstructive) or intermediate/major (≥1%; orthopedic, abdominal, vascular, hernia repair, major urological and gynecological) according to the European Society of Cardiology guidelines on cardiovascular assessment and management of patients undergoing non-cardiac surgery (18).

According to preoperative blood counts and WHO criteria (14,19), patients were stratified into three categories: anemia (hemoglobin value <120 g/L for women; <130 g/L for men), normal hemoglobin levels, and polycythemia (hemoglobin levels exceeding 165 g/L for men and 160 g/L for women; in line with the 2022 WHO diagnostic criteria for PV) (14). Complete blood counts were determined with Siemens Advia 120, 2100, and 2120i (Siemens Medical Solutions Diagnostics Pte Ltd, Swords, Ireland).

The study was performed according to the Declaration of Helsinki and was approved by the Ethics Committee of the General Hospital of Šibenik-Knin County. Due to the retrospective design of the study, informed consent was not considered mandatory.

### Statistical analysis

The Shapiro-Wilk's test was used to assess the data distribution. Differences in categorical variables were assessed with a χ^2^ test or Fisher exact test. The Kruskal-Wallis test was used to assess the differences in continuous variables across different patient categories. The primary outcome was a 30-day postoperative composite event consisting of death, arterial (myocardial infarction, transitory ischemic attack, ischemic stroke, or peripheral arterial occlusion) or venous thrombotic event (VTE; pulmonary embolism or deep vein thrombosis), the need for postoperative red blood cell transfusion, or major bleeding. The latter was classified according to the International Society on Thrombosis and Hemostasis as fatal bleeding, bleeding event with a drop in hemoglobin of ≥20 g/L, or symptomatic bleeding in a critical organ such as the retroperitoneum, in the intracranial, intraocular, intra-articular, intraspinal, or pericardial space, or intramuscularly with a secondary compartment syndrome (20). A receiver operating curve (ROC) analysis with the composite outcome as a classification variable was used to determine the optimal cut-off values of ASA, aCCI, and different blood cell count components. Multivariate analyses were performed with logistic regression analysis. *P* values <0.05 were considered statistically significant. *P* values for three simultaneous comparisons were Bonferroni corrected and set at <0.017. Statistical analysis was performed with MedCalc Statistical Software® (Ostend, Belgium, v.23.1.5).

## Results

### Patients' characteristics

The outcomes of 1196 elective surgery procedures were evaluated (minor, n = 431, 36%; intermediate/major, n = 765, 64%). A complete list with the types of surgery procedures is shown in Supplemental Table 1(Supplementary Table 1).

There were 617 (51.6%) men in the sample. The median age was 65 years (range 18-91), the median aCCI was 4 points (range 0-17), and the median ASA score was 2 (range 0-4). Preoperatively, the median hemoglobin level was 141 g/L (range 80-182). A total of 1000 (83.6%) patients had normal hemoglobin levels, 152 (12.7%) had anemia, and 44 (3.7%) had polycythemia. A minority of patients with polycythemia (3/44, 6.8%) was preoperatively evaluated by a hematologist (including Janus kinase 2 [*JAK2*] mutational testing). Patients with polycythemia were the youngest, were more frequently men and smokers, and had the highest hematocrit levels, erythrocyte and leukocyte counts, and the lowest frequency of prior VTE. Patients with anemia were the oldest, had the highest cumulative comorbidity burden (aCCI), most frequently had cancer and prior VTE, and had the lowest hematocrit and erythrocyte counts; these patients also most often used anticoagulants and underwent intermediate/major surgery procedures (Table 1).

**Table 1:** Patients' preoperative characteristics stratified according to preoperative hemoglobin categories (World Health Organization criteria for anemia and polycythemia vera). The χ^2^, Fisher exact test, and Kruskal-Wallis test were used*

Variable	Anemia (n = 152, 12.7%) (1)	Normal hemoglobin (n = 1000, 83.6%) (2)	Polycythemia (n = 44, 3.7%) (3)	P value
**Age, years (median, range)**	73 (21-91)	64 (18-91)	63 (22-88)	<0.001^†‖^ (1 vs 2,3)
**Sex, female (%)**	72 (47.4)	503 (50.3)	4 (9.1)	<0.001†‡ (3 vs 1,2)
**Leukocytes, ×10^9^/L (median, range)**	6.6 (2-15.7)	7.1 (1-9)	7.5 (3.9-14.9)	0.021^‖^ (1 vs 2,3)
**Platelets, ×10^9^/L (median, range)**	224.5 (54-456)	223.5 (55-580)	209 (87-378)	0.250^‖^
**Erythrocyte counts, ×10^12^/L (median, range)**	4.03 (1.32-5.14)	4.59 (1.0-6.03)5	5.46 (4.5-5.92)	<0.001^†‖^ (1 vs 2,3; 2 vs 3)
**Hemoglobin, g/L (median, range)**	115.5 (80-129)	143 (120-165)	170 (161-182)	<0.001^†‖^ (1 vs 2,3; 2 vs 3)
**Hematocrit, % (median, range)**	35.2 (24.1-46.4)	42.3 (13.9-44.4)	50.3 (40.4-54.1)	<0.001^†^‖ (1 vs 2,3; 2 vs 3)
**Arterial hypertension (%)**	89 (58.6)	447 (44.7)	26 (59.1)	0.001^†‡^ (1 vs 2)
**Diabetes mellitus (%)**	28 (18.4)	140 (14)	6 (13.6)	0.349^‡^
**Dementia (%)**	1 (0.7)	1 (0.1)	0	0.281^§^
**Cancer (%)**	71 (46.7)	259 (25.9)	9 (20.5)	<0.001^†‡^ (1 vs 2,3)
**COPD (%)**	8 (5.3)	37 (3.7)	3 (6.8)	0.412^§^
**Prior IM (%)**	11 (7.2)	46 (4.6)	3 (6.8)	0.327^§^
**Prior ICV/TIA (%)**	7 (4.6)	22 (2.2)	3 (6.8)	0.051^§^
**Prior VTE (%)**	12 (7.9)	32 (3.2)	0	0.007^†§^ (1 vs 2,3)
**Smoking, n = 1024 (%)**	15 (12.2)	230 (26.7)	15 (38.5)	<0.001^†‡^ (1 vs 2,3)
**Anticoagulants (%)**	25 (16.4)	65 (6.5)	4 (9.1)	<0.001^†§^ (1 vs 2)
**Antiplatelets (%)**	17 (11.2)	90 (9)	5 (11.4)	0.618^‡^
**Surgery risk:**				0.021^‡^ (1 vs 2,3)
**minor**	41 (27)	370 (63)	20 (45.5)	
**intermediate/major**	111 (73)	630 (37)	24 (54.5%)	
**aCCI (median, range)**	6 (0-14)	4 (0-17)	4 (0-11)	<0.001^†‖^ (1 vs 2,3)
**ASA (median, range)**	2 (1-4)	2 (0-4)	2 (1-3)	<0.001^†‖^ (1↑ vs 2, 3)
**Deaths (%)**	1 (0.7)	1 (0.1)	0	0.281^§^
**Thrombosis (%)**	1 (0.7)	3 (0.3)	0	0.718^§^
**Bleeding (%)**	12 (7.9)	65 (6.5)	8 (18.2)	0.011^†‡^ (1,2 vs 3)
**Postoperative red blood cell transfusion (%)**	7 (4.6)	11 (1.1)	0	<0.001^†§^ (1 vs 3)
**Composite outcome (%)**	14 (9.2)	69 (6.9)	8 (18.2)	0.016^†§^ (2 vs 3)
				

*Abbreviations: COPD – chronic obstructive pulmonary disorder; MI – myocardial infarction; TIA/ICV – transitory ischemic attack/ischemic cerebrovascular incident; VTE – venous thromboembolism; aCCI – age-adjusted Charlson Comorbidity Index; ASA – American Society of Anesthesiologists.

†Significant p value was Bonferroni corrected and set at <0.017.

‡χ^2^ test was used to analyze differences in categorical variables among the three patient groups.

§Fisher exact test was used as an alternative to χ^2^ test when assessing differences in categorical variables when one or more cell counts were less than 5.

‖Kruskal-Wallis test was used for comparisons of continuous variables among the three independent patient groups.

### Associations of preoperative polycythemia with postoperative outcomes

The composite outcome was recorded in 91 surgery procedures (7.6%) and was more frequent in patients with polycythemia (18.2%) than in those with normal hemoglobin (6.9%) or anemia (9.2%; *P* = 0.016). There were two deaths (0.16%), four thrombotic events (0.3%), 85 (7.1%) bleeding events, and 18 (1.5%) red blood cell transfusions. Multiple patients experienced two or more postoperative events (ie, bleeding, a drop in hemoglobin level, and red blood cell transfusion). Each adverse postoperative event is summarized in Supplemental Table 2(Supplementary material 2).

**Figure 1:**
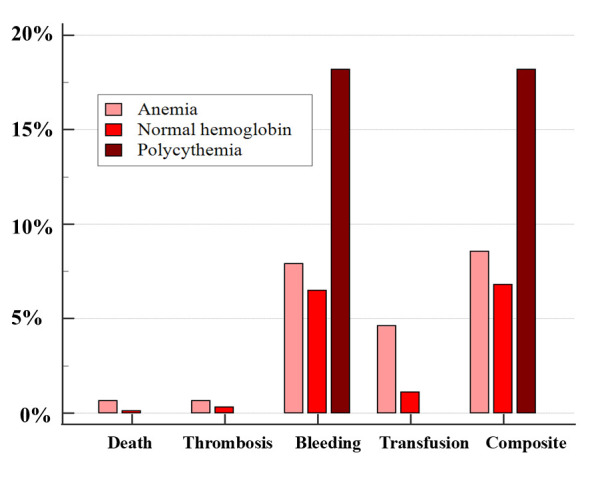
Postoperative 30-day death, thrombosis, bleeding, red blood cell transfusion, and a composite outcome (consisting of death, thrombosis, bleeding, red blood cell transfusion rate) according to preoperative hemoglobin categories (World Health Organization criteria for anemia and polycythemia vera).

Patients with polycythemia had a postoperative bleeding event (18.2%) more frequently than patients with normal hemoglobin levels (6.5%) or those with anemia (7.9%; *P* = 0.011). Patients with preoperative anemia more frequently required postoperative blood transfusions (4.6% vs 1.1% vs 0% for patients with anemia, normal hemoglobin, and polycythemia, respectively; *P* = 0.003) (Figure 1). Deaths and thrombotic events were not recorded in patients with polycythemia.

A higher occurrence of the composite outcome was also significantly univariately associated with a higher ASA score (>2) (n = 1152, 55/432, 12.7% vs 32/720, 4.4%; *P* < 0.001) and a higher aCCI (>3) (63/599, 10.5% vs 28/597, 4.7%; *P* = 0.001), but not with other clinical and laboratory variables. Both higher ASA and higher aCCI were associated with a higher incidence of postoperative bleeding and the need for red blood cell transfusions (*P* < 0.001 for all).

The associations of preoperative polycythemia with the postoperative composite outcome and bleeding were then tested in a series of multivariate logistic regression analyses adjusted for clinically meaningful variables: surgery risk, sex, aCCI, ASA, and the use of antiplatelets and anticoagulants. Preoperative polycythemia, surgery type, and higher ASA and CCI scores remained associated with a higher incidence of the composite outcome and postoperative bleeding independently of each other (Table 2). In an identical logistic regression model, preoperative polycythemia was not independently associated with the need for postoperative red blood cell transfusion (*P* = 0.997), whereas preoperative anemia (odds ratio [OR] 3.93; *P* = 0.002), high aCCI (OR 4.83; *P* = 0.041), and high ASA score (OR 4.83; *P* = 0.005) were independent predictors of the need for postoperative red blood cell transfusion. Multivariate associations of polycythemia with death and thrombosis were not assessed due to a low number of events and the subsequent lack of statistical power.

**Table 2:** Multivariate logistic regression models for a 30-day postoperative composite outcome and bleeding, adjusted for clinically meaningful variables*

	30-day composite outcome			30-day bleeding		
Variable	OR	95% CI	P value*	OR	95% CI	P value^†^
**Polycythemia**	2.84	1.21-6.63	0.015	3.04	1.02-2.86	0.010
Intermediate/major surgery	1.77	1.07-2.92	0.025	1.71	0.58-2.12	0.039
ASA score >2	2.82	1.73-4.58	<0.001	2.60	1.58-4.28	0.002
aCCI>3	1.72	1.03-2.88	0.037	1.75	1.02-2.97	0.038
Anticoagulants	0.71	0.31-1.59	0.408	0.79	0.35-1.78	0.572
Antiplatelets	1.37	0.52-3.57	0.520	1.36	0.52-3.55	0.529
Sex	0.80	0.45-1.25	0.377	0.81	0.50-1.32	0.408

*Abbreviations: OR – odds ratio, CI – confidence interval, ASA – American Society of Anesthesiologists, aCCI – age-adjusted Charlson Comorbidity Index.

†Significant *P* value set at <0.050.

Finally, we tested for potential risk factors of inferior postoperative outcomes specifically in polycythemic patients. Interestingly, none of the smokers experienced the composite outcome and bleeding (0 vs 46.9%; *P* = 0.022 for both analyses). Leukocyte or platelet counts (stratified as low, high, or median) were not associated with either an inferior composite outcome or postoperative bleeding, either in the entire cohort or in patients with polycythemia (*P* > 0.050 for all). No alternative threshold using a ROC analysis could be established to identify different potential cut-off levels of preoperative blood counts to be associated with inferior postoperative outcomes in the entire cohort or in patients with polycythemia.

## Discussion

Interestingly, even though in our study at baseline patients with anemia seemed as the risky ones, preoperative polycythemia was in fact associated with a higher rate of the composite postoperative outcome and bleeding. The independent effect of polycythemia on the composite outcome and postoperative bleeding was also confirmed in multivariate analyses and was independent of the surgery risk, comorbidity burden, or preoperative patient physical status as assessed by the ASA score. The pathophysiological mechanism underlying this association remains elusive. Notably, patients with PV have high postoperative hemorrhage rates, despite an adequate control of blood cell counts (6-8). General risk factors in MPNs associated with pathological bleedings include the use of aspirin and anticoagulants, disease phenotype (myelofibrosis>PV>essential thrombocythemia), extreme thrombocytosis (ie, >1 × 1000-1500 × 10^9^/L), leukocytosis, higher *JAK2* allele burden, the presence of calreticulin mutations, arterial hypertension, and male sex (21-23). Interestingly, patients with polycythemia in this study were predominantly male, with higher erythrocyte and leukocyte counts, and higher hematocrit; these characteristics align with those from prior reports of patients with SP (11-13,24). Another possible explanation may be smoking, which was more prevalent in patients with polycythemia than in those with anemia but was at a similar frequency as in patients with normal hemoglobin levels. Smoking has been consistently associated with inferior postoperative outcomes, as it causes poor wound healing, increases heart rate, blood pressure, and peripheral vascular resistance, reduces oxygen transport, and inhibits platelet aggregation (25-28). Moreover, smoking may also cause polycythemia. Therefore, smoking, higher blood cell counts, increased blood viscosity, and secondary tissue hypoperfusion may have been responsible for the inferior postoperative outcomes. However, in univariate analyses, blood cell counts were not associated with the composite outcome or bleeding. Surprisingly, smoking was protective in patients with polycythemia; this somewhat counterintuitive observation is probably a statistical artifact due to the low number of patients with polycythemia included. For this reason, studies including a larger number of patients with polycythemia are needed to unravel the exact pathophysiologic mechanisms behind the association of preoperative polycythemia with inferior postoperative outcomes.

In recent years, the number of investigations into PV increased significantly worldwide, mainly due to the lower hemoglobin thresholds for its diagnosis (14). For example, the Croatian Cooperative Group for Hematologic Diseases has shown that 7.7% of the first referrals to outpatient hematologic clinics in Croatia were due to polycythemia (29). Indeed, the proportion of patients with polycythemia in this study (3.7%) was similar to that in a Canadian study (15). Prior studies investigating this issue were performed on a smaller number of patients (ie, 24-100 polycythemic patients) and had a very small number of events, which precluded firm conclusions; however, patients with SP had lower transfusion requirements (9) and higher thrombotic risk compared with those with PV (10). Considering the association of SP with an increased cardiovascular risk in the general population (10-13) and the recent modification of the WHO diagnostic criteria for PV (14), we found it relevant and timely to address this issue in a larger cohort of patients.

Notably, preoperative polycythemia was not associated with the occurrence of postoperative thrombosis and death; however, due to the small number of these events no firm conclusions can be drawn. Generally, a small number of postoperative deaths and thrombotic events, and the fact that none of the patients with polycythemia experienced thrombosis or death may be reassuring. Additionally, none of the patients with polycythemia required postoperative blood transfusion. This observation is also relevant considering the known potential harms of transfusion and the fact that it does not compensate for the poor perioperative outcomes conferred by anemia (3,30,31).

Finally, ASA classification and aCCI were both univariately and multivariately associated with adverse postoperative outcomes. Both of these scoring systems cumulatively and comprehensively assess patient fitness and comorbidities. Considering that cancer and prior venous thrombosis were the only individual patient comorbidities associated with inferior postoperative outcomes, these observations again highlight the importance of a timely and comprehensive preoperative assessment of the cumulative patient comorbidity burden to optimize postoperative outcomes.

Limitations of this study are the retrospective single-center design with its intrinsic biases, small number of specific events, and the fact that some of them may have not been captured in the medical documentation. Additionally, the median preoperative hemoglobin level of patients with polycythemia was 170 g/L, and the highest one was 182 g/L. Therefore, it is unclear what the postoperative outcomes would have been if only patients with very high hemoglobin levels had been included. Also, some of the patients with polycythemia may have had PV. However, three of these patients were evaluated by a hematologist, who diagnostically excluded PV. Also, PV is a very rare disease (the annual incidence in the European Union is 0.4-28 per 100 000) (32), and thus it is reasonable to conclude that not many PV patients have been missed. Finally, due to the retrospective design of the study, the causality of polycythemia with inferior postoperative outcomes cannot be inferred due to the possibility of residual confounding.

Despite these limitations, this study for the first time provides an important signal regarding possible inferior postoperative outcomes in unselected patients presenting preoperatively with WHO-defined polycythemia. Therefore, patients with polycythemia need to be carefully preoperatively assessed. Future studies are warranted to validate the presented results in different demographic populations and to investigate whether these patients might benefit from pre-treatment with phlebotomies or the optimization of antiplatelet or anticoagulant therapy.

## References

[1] Kumar A Srivastava U Role of routine laboratory investigations in preoperative evaluation. J Anaesthesiol Clin Pharmacol 2011 27 174 9 10.4103/0970-9185.81824 21772675 PMC3127294

[2] Bock M Fritsch G Hepner DL Preoperative laboratory testing. Anesthesiol Clin 2016 34 43 58 10.1016/j.anclin.2015.10.005 26927738

[3] Lin Y Preoperative anemia-screening clinics. Hematology (Am Soc Hematol Educ Program) 2019 2019 570 6 10.1182/hematology.2019000061 31808909 PMC6913451

[4] Krecak I Lucijanic M Verstovsek S Advances in risk stratification and treatment of polycythemia vera and essential thrombocythemia. Curr Hematol Malig Rep 2022 17 155 69 10.1007/s11899-022-00670-8 35932395

[5] Pemmaraju N Gerds AT Yu J Parasuraman S Shah A Xi A Thrombotic events and mortality risk in patients with newly diagnosed polycythemia vera or essential thrombocythemia. Leuk Res 2022 115 106809 10.1016/j.leukres.2022.106809 35220060

[6] Ruggeri M Rodeghiero F Tosetto A Castaman G Scognamiglio F Finazzi G Postsurgery outcomes in patients with polycythemia vera and essential thrombocythemia: a retrospective survey. Blood 2008 111 666 71 10.1182/blood-2007-07-102665 17909074

[7] Szuber N Olney HJ Dagenais Bellefeuille S Tangua M Shehabeldeen A Ahmed S Perioperative outcomes in patients with myeloproliferative neoplasms: a multicentric analysis of 354 surgical procedures. Blood Vessels Thromb Haemost 2024 1 100026 10.1016/j.bvth.2024.100026 PMC12320445 40765935

[8] Labaran LA Amin R Sequeira S Puvanesarajah V Haug E Rao SS Does polycythemia vera increase risk of postoperative complications following primary total joint arthroplasty? A retrospective matched control cohort study of 6932 polycythemia vera patients. J Arthroplasty 2020 35 6S S133 7 10.1016/j.arth.2019.10.050 31776052

[9] Lubarsky DA Gallagher CJ Berend JL Secondary polycythemia does not increase the risk of perioperative hemorrhagic or thrombotic complications. J Clin Anesth 1991 3 99 103 10.1016/0952-8180(91)90004-7 2039651

[10] Szuber N Harnois M Delage R Sirhan S Busque L Higher rates of perioperative thrombosis and distinct management practices in secondary erythrocytosis versus polycythemia vera. Blood 2022 140 Supplement 1 5661 566 10.1182/blood-2022-170026

[11] Nguyen E Harnois M Busque L Sirhan S Assouline S Chamaki I Phenotypical differences and thrombosis rates in secondary erythrocytosis versus polycythemia vera. Blood Cancer J 2021 11 75 10.1038/s41408-021-00463-x 33859172 PMC8050282

[12] Krečak I Holik H Zekanović I Morić Perić M Marketin T Coha B Thrombotic risk in secondary polycythemia resembles low-risk polycythemia vera and increases in specific subsets of patients. Thromb Res 2022 209 47 50 10.1016/j.thromres.2021.11.025 34864474

[13] Wouters HJCM Mulder R van Zeventer IA Schuringa JJ van der Klauw MM van der Harst P Erythrocytosis in the general population: clinical characteristics and association with clonal hematopoiesis. Blood Adv 2020 4 6353 63 10.1182/bloodadvances.2020003323 33351130 PMC7757002

[14] Khoury JD, Solary E, Abla O, Akkari Y, Alaggio R, Apperley JF, et al. The 5th edition of the World Health Organization Classification of Haematolymphoid Tumours: Myeloid and Histiocytic/Dendritic Neoplasms. Leukemia. 2022;36(7):1703-1719. 10.1038/s41375-022-01613-1 PMC9252913 35732831

[15] Busque L Porwit A Day R Olney HJ Leber B Éthier V Laboratory investigation of myeloproliferative neoplasms (mPNs): recommendations of the Canadian Mpn Group. Am J Clin Pathol 2016 146 408 22 10.1093/ajcp/aqw131 27686169

[16] Charlson ME Pompei P Ales KL MacKenzie CR A new method of classifying prognostic comorbidity in longitudinal studies: development and validation. J Chronic Dis 1987 40 373 83 10.1016/0021-9681(87)90171-8 3558716

[17] Doyle DJ, Hendrix JM, Garmon EH. American Society of Anesthesiologists Classification. 2023. In: StatPearls [Internet]. Treasure Island (FL): StatPearls Publishing; 2024.

[18] Halvorsen S Mehilli J Cassese S Hall TS Abdelhamid M Barbato E 2022 ESC Guidelines on cardiovascular assessment and management of patients undergoing non-cardiac surgery. Eur Heart J 2022 43 3826 924 10.1093/eurheartj/ehac270 36017553

[19] Cappellini MD Motta I Anemia in clinical practice—definition and classification: does hemoglobin change with aging? Semin Hematol 2015 52 261 9 10.1053/j.seminhematol.2015.07.006 26404438

[20] Schulman S Kearon C Subcommittee on Control of Anticoagulation of the Scientific and Standardization Committee of the International Society on Thrombosis and Haemostasis Definition of major bleeding in clinical investigations of antihemostatic medicinal products in non-surgical patients. J Thromb Haemost 2005 3 692 4 10.1111/j.1538-7836.2005.01204.x 15842354

[21] Landolfi R Cipriani MC Novarese L Thrombosis and bleeding in polycythemia vera and essential thrombocythemia: pathogenetic mechanisms and prevention. Best Pract Res Clin Haematol 2006 19 617 33 10.1016/j.beha.2005.07.011 16781491

[22] Nicol C Lacut K Pan-Petesch B Lippert E Ianotto JC Hemorrhage in essential thrombocythemia or polycythemia vera: epidemiology, location, risk factors, and lessons learned from the literature. Thromb Haemost 2021 121 553 64 10.1055/s-0040-1720979 33186994

[23] Zwicker JI Paranagama D Lessen DS Colucci PM Grunwald MR Hemorrhage in patients with polycythemia vera receiving aspirin with an anticoagulant: a prospective, observational study. Haematologica 2022 107 1106 10 10.3324/haematol.2021.279032 34162181 PMC9052904

[24] Krečak I, Holik H, Morić Perić M, Zekanović I, Coha B, Gverić-Krečak V, et al. High platelet-to-lymphocyte ratio may differentiate polycythemia vera from secondary polycythemia. Wien Klin Wochenschr. 20222;134(11-12):483-486. 10.1007/s00508-022-02027-w 35391561

[25] Fan Chiang YH Lee YW Lam F Liao CC Chang CC Lin CS Smoking increases the risk of postoperative wound complications: A propensity score-matched cohort study. Int Wound J 2023 20 391 402 10.1111/iwj.13887 35808947 PMC9885463

[26] Schmid M Sood A Campbell L Kapoor V Dalela D Impact of smoking on perioperative outcomes after major surgery. Am J Surg 2015 210 221 229.e6 10.1016/j.amjsurg.2014.12.045 25980408

[27] Yoshikawa R Katada J Effects of active smoking on postoperative outcomes in hospitalised patients undergoing elective surgery: a retrospective analysis of an administrative claims database in Japan. BMJ Open 2019 9 e029913 10.1136/bmjopen-2019-029913 31575535 PMC6797353

[28] Wong J Lam DP Abrishami A Chan MT Chung F Short-term preoperative smoking cessation and postoperative complications: a systematic review and meta-analysis. Can J Anaesth 2012 59 268 79 10.1007/s12630-011-9652-x 22187226

[29] Vodanovic M, Pulanic D, Radman I, Krecak I, Holik H, Racetin A. The analysis of the first hematological consultations in Croatian hematology outpatient clinics – a KROHEM study. In, EHA Library, 2021. Abstract PB1605.

[30] Ferraris VA Davenport DL Saha SP Austin PC Zwischenberger JB Surgical outcomes and transfusion of minimal amounts of blood in the operating room. Arch Surg 2012 147 49 55 10.1001/archsurg.2011.790 22250113

[31] Ferraris VA Hochstetler M Martin JT Mahan A Saha SP Blood transfusion and adverse surgical outcomes: The good and the bad. Surgery 2015 158 608 17 10.1016/j.surg.2015.02.027 26032824

[32] Moulard O Mehta J Fryzek J Olivares R Iqbal U Mesa RA Epidemiology of myelofibrosis, essential thrombocythemia, and polycythemia vera in the European Union. Eur J Haematol 2014 92 289 97 10.1111/ejh.12256 24372927

