# Supplementary material for: Preoperative polycythemia may be associated with inferior postoperative outcomes: a retrospective study
Source: Croat Med J. 2025 Jun;66(3):186–93. doi: 10.3325/cmj.2025.66.186 (PMC12246972; doi:10.3325/cmj.2025.66.186)
Supplement: Supplementary Table 1 [file CroatMedJ_66_s001.pdf]

Supplementary Table S1. A list of surgery procedures.

**MINOR SURGERY (n=431, 36%)**	**INTERMEDIATE and MAJOR SURGERY (n=765, 64%)**
Gynecology (n=3, 0.7%)	Gynecology (n=97, 12.7%)
Urology (n=199, 46.2%)	Abdomen (n=586, 76.6%)
Breast (n=108, 25.1%)	Orthopedic/trauma (n=81, 10.6%)
Orthopedic (n=52, 12.1%)	Ear, nose and throat (n=1, 0.1%)
Ear, nose and throat (n=55, 12.8%)	
Superficial skin (n=11, 2.6%)	
Varicose veins (n=3, 0.7%)	
