# Supplementary material for: Preoperative polycythemia may be associated with inferior postoperative outcomes: a retrospective study
Source: Croat Med J. 2025 Jun;66(3):186–93. doi: 10.3325/cmj.2025.66.186 (PMC12246972; doi:10.3325/cmj.2025.66.186)
Supplement: Supplementary Table 2 [file CroatMedJ_66_s002.pdf]

Supplementary Table S2. Patient-level data regarding the type of 30-day adverse postoperative outcomes.

Patient number	Hemoglobin category	Surgery risk; type of procedure	Adverse postoperative outcome
784	Normal	Intermediate/major; hemicolectomy	Death
797	Anemia	Intermediate/major; short bowel resection	Death
217	Anemia	Intermediate/major; hernia repair	Ischemic stroke
222	Normal	Intermediate/major; sygmoid colon resection	Deep vein thrombosis and pulmonary embolism
418	Normal	Low; meniscetomy	Pulmonary embolism
931	Normal	Intermediate/major; miomectomy	Ischemic stroke
53	Anemia	Low; transurethral bladder resection	Hematuria and drop in hemoglobin level ≥20g/L
165	Polycythemia	Intermediate/major; hip replacement	Local hematoma and drop in hemoglobin level ≥20g/L
220	Anemia	Intermediate/major; hernia repair	Rectal bleeding, drop in hemoglobin level ≥20g/L, and the need for red blood cell transfusions
256	Normal	Intermediate/major; subtotal colectomy	Intraabdominal bleeding and drop in hemoglobin level ≥20g/L
289	Normal	Intermediate/major; colon cancer resection	Intraabdominal bleeding, drop in hemoglobin level ≥20g/L, and the need for red blood cell transfusion
310	Normal	Intermediate/major; colon cancer resection	Intraabdominal bleeding, drop in hemoglobin level ≥20g/L, and the need for red blood cell transfusion
358	Polycythemia	Intermediate/major; hip replacement	Drop in hemoglobin level≥20g/L
360	Normal	Intermediate/major; gastrectomy	Intraabdominal hematoma, drop in hemoglobin level ≥20g/L
364	Normal	Intermediate/major; hip replacement	Local hematoma, drop in hemoglobin level ≥20g/L, and the need for red blood cell transfusion
367	Normal	Low; transurethral bladder resection	Hematuria and drop in hemoglobin level ≥20g/L
438	Normal	Intermediate/major; colon cancer resection - hemicolectomy	Intraabdominal bleeding, drop in hemoglobin level ≥20g/L, and the need for red blood cell transfusion

446	Normal	Low; transurethral prostate resection	Hematuria and drop in hemoglobin level ≥20g/L
477	Normal	Intermediate/major; hernia repair	Drop in hemoglobin level ≥20g/L
487	Normal	Low; transurethral bladder resection	Hematuria and drop in hemoglobin level ≥20g/L
506	Normal	Low; partial mastectomy	Local hematoma, drop in hemoglobin level ≥20g/L, and the need for red blood cell transfusion
537	Normal	Intermediate/major; hip replacement	Drop in hemoglobin level≥20g/L
543	Normal	Low; partial mastectomy	Drop in hemoglobin level ≥20g/L
563	Normal	Intermediate/major; hip replacement	Drop in hemoglobin level≥20g/L
574	Normal	Intermediate/major; colon cancer resection - hemicolectomy	Drop in hemoglobin level≥20g/L
585	Anemia	Intermediate/major; intraabdominal adhesiolysis	Drop in hemoglobin level≥20g/L
592	Anemia	Intermediate/major; knee replacement	Drop in hemoglobin level≥20g/L
594	Anemia	Intermediate/major; colon cancer resection - hemicolectomy	Drop in hemoglobin level≥20g/L
676	Normal	Low; transurethral bladder resection	Hematuria and drop in hemoglobin level ≥20g/L
679	Normal	Intermediate/major; hip replacement	Drop in hemoglobin level≥20g/L
704	Normal	Intermediate/major; hysterectomy	Drop in hemoglobin level≥20g/L
710	Anemia	Intermediate/major; hiatal hernia repair	Gastrointestinal bleeding, drop in hemoglobin level ≥20g/L, and the need for red blood cell transfusion
724	Normal	Low; transurethral bladder resection	Drop in hemoglobin level≥20g/L
728	Normal	Intermediate/major; hip replacement	Drop in hemoglobin level≥20g/L
751	Anemia	Intermediate/major; —— hemicolectomy	Gastrointestinal bleeding, drop in hemoglobin level ≥20g/L, and the need for red blood cell transfusion
776	Polycythemia	Intermediate/major; laparotomy and cholecystectomy	Drop in hemoglobin level≥20g/L
782	Anemia	Intermediate/major; choledocus surgery	Gastrointestinal bleeding, drop in hemoglobin level ≥20g/L, and the need for red blood cell transfusion

784	Normal	Intermediate/major; hemicolectomy	Drop in hemoglobin level≥20g/L
788	Normal	Low; partial mastectomy	Local hematoma and drop in hemoglobin level ≥20g/L
789	Normal	Intermediate/major; hemicolectomy	Drop in hemoglobin level≥20g/L
797	Anemia	Intermediate/major; short bowel resection	Need for red blood cell transfusion
802	Normal	Intermediate/major; hip replacement	Drop in hemoglobin level≥20g/L
807	Normal	Intermediate/major; hip replacement	Drop in hemoglobin level≥20g/L
819	Normal	Intermediate/major; histerectomy	Drop in hemoglobin level≥20g/L
821	Anemia	Low; transurethral bladder resection	Hematuria and drop in hemoglobin level ≥20g/L
825	Normal	Intermediate/major; ventral hernia repair	Drop in hemoglobin level≥20g/L
828	Anemia	Low; transurethral bladder resection	Hematuria and drop in hemoglobin level ≥20g/L
834	Normal	Intermediate/major; hemicolectomy	Drop in hemoglobin level≥20g/L
841	Polycythemia	Intermediate/major; ventral and inguinal hernia repair	Drop in hemoglobin level≥20g/L
845	Normal	Intermediate/major; cholecytectomy	Drop in hemoglobin level≥20g/L
847	Normal	Intermediate/major; hemorrhoid surgery	Drop in hemoglobin level≥20g/L
855	Normal	Low; transurethral bladder resection	Drop in hemoglobin level≥20g/L
856	Normal	Intermediate/major; hemicolectomy	Drop in hemoglobin level≥20g/L
863	Normal	Intermediate/major; cholecytectomy	Drop in hemoglobin level≥20g/L
870	Normal	Intermediate/major; ventral and inguinal hernia repair	Drop in hemoglobin level≥20g/L
881	Anemia	Intermediate/major; hemicolectomy	Drop in hemoglobin level≥20g/L
892	Normal	Intermediate/major; hip replacement	Drop in hemoglobin level≥20g/L
897	Normal	Intermediate/major; hemicolectomy	Drop in hemoglobin level≥20g/L
903	Normal	Intermediate/major; histerectomy	Drop in hemoglobin level≥20g/L
905	Normal	Intermediate/major; histerectomy	Drop in hemoglobin level≥20g/L
914	Normal	Intermediate/major; oovariectomy	Drop in hemoglobin level≥20g/L

931	Normal	Intermediate/major; histerectomy	Drop in hemoglobin level≥20g/L
946	Normal	Low; partial mastectomy	Drop in hemoglobin level≥20g/L
951	Polycythemia	Low; transurethral bladder resection	Hematuria and drop in hemoglobin level ≥20g/L
955	Normal	Intermediate/major; hip replacement	Local hematoma, drop in hemoglobin level≥20g/L
960	Anemia	Low; transurethral bladder resection	Hematuria and drop in hemoglobin level ≥20g/L
963	Normal	Intermediate/major; knee replacement	Drop in hemoglobin level≥20g/L
973	Normal	Intermediate/major; hip replacement	Local hematoma and drop in hemoglobin level ≥20g/L
983	Polycythemia	Low; uretheral stone	Hematuria and drop in hemoglobin level ≥20g/L
991	Normal	Intermediate/major; colon cancer resection	Gastrointestinal bleeding and drop in hemoglobin level ≥20g/L
995	Normal	Low; prolapse of the uterus	Vaginal bleeding and drop in hemoglobin level ≥20g/L
996	Normal	Low; transurethral bladder resection	Hematuria and drop in hemoglobin level ≥20g/L
997	Polycythemia	Intermediate/major; knee replacement	Local hematoma and drop in hemoglobin level ≥20g/L
1003	Anemia	Low; prolapse of the uterus	Vaginal bleeding, drop in hemoglobin level ≥20g/L, and the need for red blood cell transfusion
1009	Normal	Low; transurethral prostate resection	Hematuria and drop in hemoglobin level ≥20g/L
1030	Normal	Intermediate/major; hernia repair	Drop in hemoglobin level≥20g/L
1031	Normal	Low; bladder kidney stone extraction	Hematuria and drop in hemoglobin level ≥20g/L
1033	Anemia	Low; urether stone extraction, prothesis implantation	Hematuria, drop in hemoglobin level ≥20g/L, and the need for red blood cell transfusion
1058	Normal	Intermediate/major; colon cancer resection	Gastrointestinal bleeding and drop in hemoglobin level ≥20g/L
1061	Normal	Low; transurethral prostate resection	Hematuria and drop in hemoglobin level ≥20g/L
1062	Normal	Low; transurethral prostate resection	Hematuria and drop in hemoglobin level ≥20g/L

1064	Normal	Intermediate/major; adnexectomy	Intraabdominal bleeding, drop in hemoglobin level ≥20g/L, and the need for red blood cell transfusion
1068	Normal	Intermediate/major; colon cancer resection	Intraabdominal bleeding, drop in hemoglobin level ≥20g/L, and the need for red blood cell transfusion
1092	Normal	Intermediate/major; colon cancer resection	Gastrointestinal bleeding and drop in hemoglobin level ≥20g/L
1098	Normal	Intermediate/major; colon cancer resection	Intraabdominal bleeding, drop in hemoglobin level ≥20g/L, and the need for red blood cell transfusion
1099	Normal	Intermediate/major; colon cancer resection	Gastrointestinal bleeding and drop in hemoglobin level ≥20g/L
1116	Normal	Low; transurethral prostate resection	Hematuria, drop in hemoglobin level ≥20g/L, and the need for red blood cell transfusion
1120	Normal	Intermediate/major; histerectomy	Vaginal bleeding, fall in hemoglobin level ≥20g/L
1124	Normal	Low; partial mastectomy	Local hematoma and the fall in hemoglobin level ≥20g/L
1133	Polycythemia	Intermediate/major; hip replacement	Local hematoma and the fall in hemoglobin level ≥20g/L
1144	Normal	Intermediate/major; hernia repair	Local hematoma and the fall in hemoglobin level ≥20g/L
1159	Normal	Intermediate/major; rectal cancer	Intraabdominal bleeding, drop in hemoglobin level ≥20g/L, and the need for red blood cell transfusion
1160	Normal	Low; transurethral prostate resection	Hematuria, drop in hemoglobin level ≥20g/L, and the need for red blood cell transfusion
1161	Normal	Intermediate/major; sygmoid cancer	Intermediate/major; rectal cancer
1176	Normal	Intermediate/major; colon cancer resection	Postoperative drop in hemoglobin level ≥20g/L
1193	Normal	Intermediate/major; hip replacement	Local hematoma and the fall in hemoglobin level ≥20g/L
